# Supplementary material for: Molecular dynamics ensemble refinement of the heterogeneous native state of NCBD using chemical shifts and NOEs
Source: PeerJ. 2018 Jul 4;6:e5125. doi: 10.7717/peerj.5125 (PMC6035720; doi:10.7717/peerj.5125)
Supplement: Table S1 — The number of simulated annealing (SA) cycles listed represents the number of cycles performed (for each replica) and analyzed after discarding the first 45 cycles for convergence. [file peerj-06-5125-s001.docx]

**Table S1. Summary of the MD ensembles obtained in this study.** The number of simulated annealing (SA) cycles listed represents the number of cycles performed (for each replica) and analyzed after discarding the first 45 cycles for convergence.

| **MD ensemble** | **Number of replicas** | **Number of SA cycles per replica** | **Experimental restraints used** | **Force Field** | **Starting Structure** |
| --- | --- | --- | --- | --- | --- |
| unbiased | // |  | // | CHARMM22* | 2KKJ |
| CS-1 | 1 | 320 | CS | CHARMM22* | 2KKJ |
| CS-2 | 2 | 160 | CS | CHARMM22* | 2KKJ |
| CS-4 | 4 | 80 | CS | CHARMM22* | 2KKJ |
| CS-8 | 8 | 40 | CS | CHARMM22* | 2KKJ |
| NOE-1 | 1 | 320 | NOEs | CHARMM22* | 2KKJ |
| NOE-2 | 2 | 160 | NOEs | CHARMM22* | 2KKJ |
| NOE-4 | 4 | 80 | NOEs | CHARMM22* | 2KKJ |
| CS-NOE-1 | 1 | 320 | NOEs | CHARMM22* | 2KKJ |
| CS-NOE-2 | 2 | 160 | CS and NOEs | CHARMM22* | 2KKJ |
| CS-NOE-2-1ZOQ | 2 | 160 | CS and NOEs | CHARMM22* | 1ZOQ |
| CS-NOE-4 | 4 | 80 | CS and NOEs | CHARMM22* | 2KKJ |
| CS-NOE-4-C22 | 4 | 80 | CS and NOEs | CHARMM22 | 2KKJ |
| CS-NOE-8 | 8 | 40 | CS and NOEs | CHARMM22* | 2KKJ |
