# Supplementary material for: Molecular dynamics ensemble refinement of the heterogeneous native state of NCBD using chemical shifts and NOEs
Source: PeerJ. 2018 Jul 4;6:e5125. doi: 10.7717/peerj.5125 (PMC6035720; doi:10.7717/peerj.5125)

Figure S1. Cross-validation of unbiased and CS-restrained ensembles using NOE measurements. We calculated the total number of NOE violations in the unbiased and CS-restrained ensembles. We used 455 NOE-derived distance restraints (BMRB entry 16363) of which 409 are short- and 46 are long-range (i.e. separated by more than 4 residues). In addition to the total number of violations (all), we also separated the violations into different categories depending on their magnitude (0.5Å-1Å, 1Å-2Å or greater than 2Å) and whether they are short (0–4 residues apart) or long-range (more than 4 residues apart).

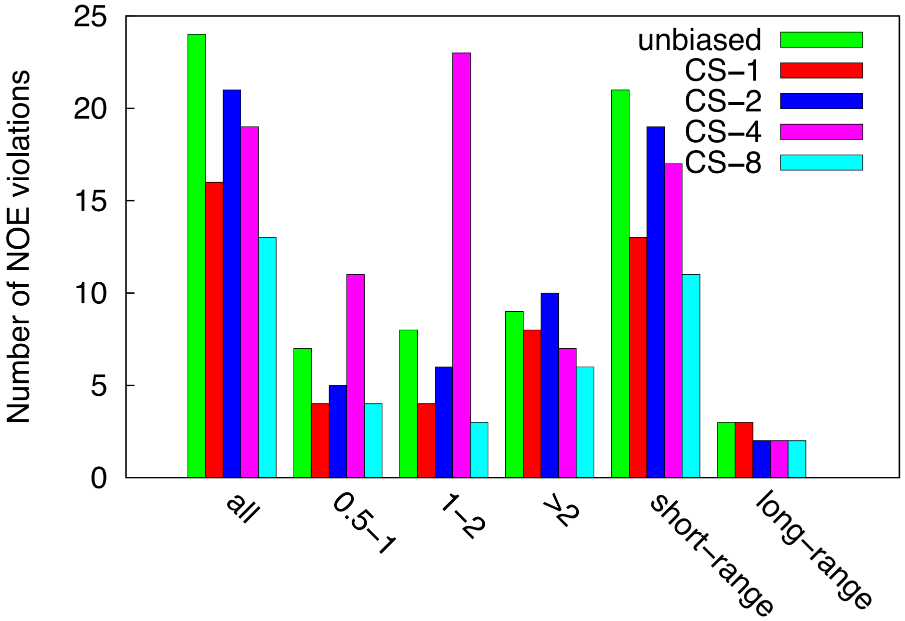

Supplement: Figure S1 — We calculated the total number of NOE violations in the unbiased and CS-restrained ensembles. We used 455 NOE-derived distance restraints (BMRB entry 16363) of which 409 are short- and 46 are long-range (i.e., separated by more than 4 residues). In addition to the total number of violations (all), we also separated the violations into different categories depending on their magnitude (0.5Å–1Å, 1Å–2Åor greater than 2Å) and whether they are short (0–4 residues apart) or long-range (more than 4 residues apart). [file peerj-06-5125-s002.pdf]
