# Supplementary material for: Molecular dynamics ensemble refinement of the heterogeneous native state of NCBD using chemical shifts and NOEs
Source: PeerJ. 2018 Jul 4;6:e5125. doi: 10.7717/peerj.5125 (PMC6035720; doi:10.7717/peerj.5125)

Figure S2. Validation of the structural ensemble using ^{1}H methyl chemical side-chain chemical shifts. We calculated the deviation between experimental and calculated side-chain ^{1}H chemical shifts from each MD ensemble with PPM to compare with the CH3Shift predictions reported in Figure 3B. The results are shown as a function of the number of replicas used for the averaging of the simulations. The previously determined NMR structure (black) and unbiased MD simulation (green) do not involve replica averaging and are shown as horizontal lines.

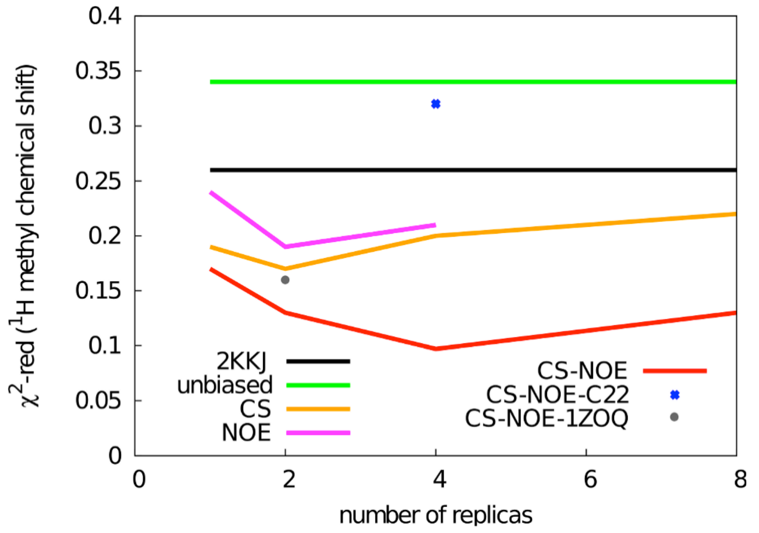

Supplement: Figure S2 — We calculated the deviation between experimental and calculated side-chain 1H chemical shifts from each MD ensemble with PPM to compare with the CH3Shift predictions reported in Fig. 3B. The results are shown as a function of the number of replicas used for the averaging of the simulations. The previously determined NMR structure (black) and unbiased MD simulation (green) do not involve replica averaging and are shown as horizontal lines. [file peerj-06-5125-s003.pdf]
