# Supplementary material for: Molecular dynamics ensemble refinement of the heterogeneous native state of NCBD using chemical shifts and NOEs
Source: PeerJ. 2018 Jul 4;6:e5125. doi: 10.7717/peerj.5125 (PMC6035720; doi:10.7717/peerj.5125)

Figure S3. Main chain RMSD of the 28 NCBD unbiased simulations started from conformations extracted from the CS-NOE-4 ensemble.

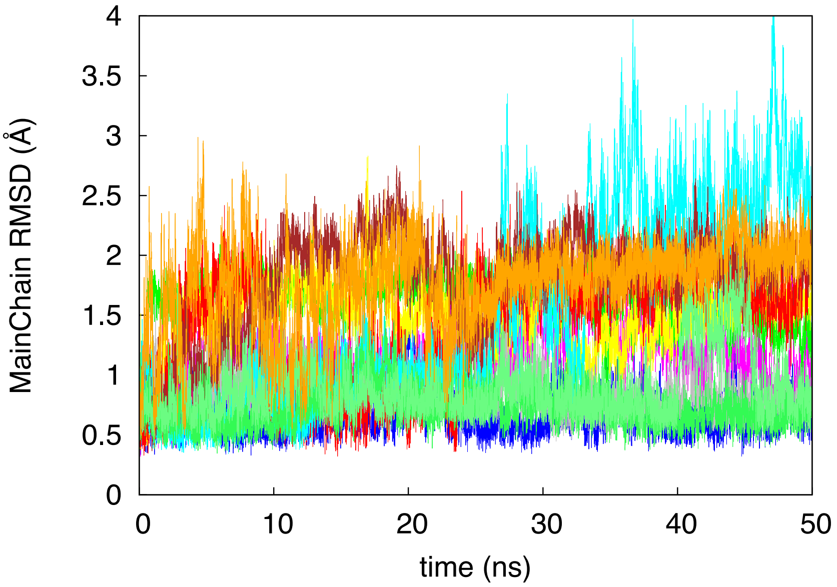

Supplement: Figure S3 [file peerj-06-5125-s004.pdf]
